# Validating Urinary Neopterin as a Biomarker of Immune Response in Captive and Wild Capuchin Monkeys

**DOI:** 10.3389/fvets.2022.918036

**Published:** 2022-07-13

**Authors:** Jordan M. Lucore, Andrew J. Marshall, Sarah F. Brosnan, Marcela E. Benítez

**Affiliations:** ^1^Department of Anthropology, University of Michigan, Ann Arbor, MI, United States; ^2^Capuchinos de Taboga Research Project, Taboga Forest Reserve, Guanacaste, Costa Rica; ^3^Program in the Environment, University of Michigan, Ann Arbor, MI, United States; ^4^Department of Ecology and Evolutionary Biology, University of Michigan, Ann Arbor, MI, United States; ^5^School for Environment and Sustainability, University of Michigan, Ann Arbor, MI, United States; ^6^Department of Psychology and Neuroscience Institute, Georgia State University, Atlanta, GA, United States; ^7^Language Research Center, Georgia State University, Atlanta, GA, United States; ^8^Department of Anthropology, Emory University, Atlanta, GA, United States

**Keywords:** immune system, immunology, physiology, primate, non-invasive sampling, health monitoring

## Abstract

Non-invasive health monitoring is advantageous for wild and captive primate populations because it reduces the need for traditional invasive techniques (i.e., anesthetization) that can be stressful and potentially harmful for individuals. The biomarker neopterin is an emerging tool in primatology to measure immune activation and immunosenescence, however, most neopterin studies have focused on catarrhine species with little comparative work examining neopterin and health in platyrrhines. To address this gap, we validated a commercially available enzyme-linked immunosorbent assay (ELISA) to measure urinary neopterin in two types of capuchin monkeys, a wild population of white-faced capuchins (*Cebus imitator*) and a socially housed captive colony of tufted capuchins (*Sapajus apella*). We analytically validated methods for measuring urinary neopterin in two capuchin populations and demonstrated that two commonly-used methods to control for urine concentration—creatinine and specific gravity (SG)—produced highly concordant results. We also biologically validated these methods by examining variation in neopterin levels based on environment (captive and wild) and age, and changes in levels associated with immune-response. We found that neopterin increased after immune perturbation (rabies vaccine booster), varied by environmental condition, and mirrored expected trends in immune system ontogeny. Our results improve understanding of the innate immune system in platyrrhine species and suggest neopterin may be useful for non-invasive health monitoring in both captive and wild primates.

## Introduction

Social animals, like non-human primates (primates hereafter), are at high risk of disease transmission due to group living (1). The growing field of ecological immunology aims to examine the relationships between ecological factors, disease, and parasite transmission in wild animal populations by monitoring health and immune function. Traditionally, health monitoring methods have required invasive interventions (e.g., blood draws) that require capture and anesthetization (2–4), stressful procedures that are difficult to conduct on arboreal species and therefore impractical for longitudinal monitoring. Non-invasive methods, such as measuring fecal parasite load (5), dipstick urinalysis (6), and visual inspection of body condition (7) allow for repetitive monitoring but often lack precision and are inadequate to monitor immune system processes associated with health and longevity.

Non-invasive sampling of the biomarker neopterin is a promising tool for monitoring health and immune status in wild primates. Urinary neopterin reflects activation of the innate immune system, the body's first line of defense against novel pathogens (8, 9). Neopterin is excreted into body fluids by macrophages and monocytes after stimulation from cytokine interferon gamma. Increase in neopterin induces T-helper 1 cells and declines when a measurable number of antibodies have been created to fight the infection (10–13). Neopterin has been used in clinical applications to monitor disease progression in humans (14) and has been found to increase in urine after HIV infection in macaque species (15–17) and respiratory infection in bonobos and chimpanzees (18, 19). Urinary neopterin has been validated and integrated into health monitoring studies in a number of primate species (*Pan troglodytes* [chimpanzees; (18–22)], *Pan paniscus* [bonobos; (18, 23)], *Macaca sylvanus* [barbary macaques; (24)] and has been shown to vary with age (18, 23, 24), differ between environments [captivity vs. wild; (20)], and respond to infection (18, 19).

Despite growing interest in utilizing neopterin as a biomarker of health and immune response in primates, these studies have focused predominantly on a small subset of primate species. In particular, to date there is comparatively little research on neopterin as a marker of health in platyrrhine primates. Since 72 species of platyrrhines are vulnerable to extinction [36 of which are endangered or critically endangered; (25)], determining whether urinary neopterin can be used as an effective biomarker of immune response in these predominantly arboreal taxa is critical for monitoring the health of remaining wild populations. Neopterin has been detected in urine in three platyrrhine families (26) and in serum in one clinical experiment with captive capuchins (27), suggesting that neopterin can be measured in these taxa. Detecting neopterin is an important first step; missing, however, is a detailed validation and comparative analyses of whether neopterin functions similarly as a biomarker of health and immune response in platyrrhine primates as it does in catarrhines. In this paper, we analytically and biologically validated urinary neopterin in two commonly studied (and con-familial) platyrrhines, tufted and white-faced capuchin monkeys.

Developing non-invasive markers of health for use in capuchin monkeys is of specific interest for several reasons. First, capuchins inhabit a wide variety of niches and exhibit a high degree of ecological flexibility (28), thereby making them a particularly interesting taxon in which to examine the effects of ecological variability on health and immune function. Second, their arboreal nature and small body sizes complicate many traditional methods of health monitoring (2, 29). Capuchins are also susceptible to intense anthropogenic encroachment due to predicted expansion of intensive agriculture in the neotropics (30) putting them at increased risk of exposure to disease through contact with humans (31, 32) and livestock (33). Validating urinary neopterin in this species would permit its use to regularly monitor health and immune function of both vulnerable wild capuchin populations and in captive facilities where capuchins are commonly used model species for biomedical and cognitive research (34).

Our first goal was to develop and analytically validate methods for measuring urinary neopterin in two populations of capuchin monkeys: a wild population of white-faced capuchins (*Cebus imitator*) in the Taboga Forest Reserve in Costa Rica and a socially housed captive colony of tufted capuchins (*Sapajus apella*) at Georgia State University. Specifically, we examined how a commercially available competitive exclusion enzyme-linked immunosorbent assay (ELISA) manufactured for human use performed measuring neopterin in capuchin urine. In addition, because neopterin levels vary with urine concentration, we compared two commonly-used methods for detecting water volume in urine in wild primates: creatinine and specific gravity (SG). Creatinine, a byproduct of metabolic pathways in muscle tissue excreted in urine, is the most common measure (16, 35–37) but its methods are less field-friendly, more expensive, and can be confounded by muscle-mass (38, 39). SG is a field-friendly, cheaper alternative. SG is the ratio of the density of the urine specimen to the density of water and increases with solute concentration, but its utility has been debated due to the limited comparative studies with creatine in both field primatology and biomedical research (36, 37, 40).

Our second goal was to biologically validate these methods by examining variation in neopterin levels as a function of immune-response, environment, and age. Specifically, we examined differences in neopterin: (1) in response to routine rabies immunization, (2) between wild and captive conditions, and (3) across age groups. To examine immune response activation, we measured neopterin levels in the captive population of *S. apella* prior to and after routine booster vaccination to confer immunity to rabies. In children, urinary neopterin had been found to increase following vaccination with the MMR vaccine (41). Thus, we predicted that urinary neopterin would increase following immunization with the rabies booster vaccine. Neopterin has also been shown to differ among animals living under different environmental conditions. Wild animals are thought to invest more in their immune systems than their captive counterparts and increased immune system investment in wild populations has been shown in birds (42) and dolphins (43). In a recent study on chimpanzees, neopterin was nearly twice as high in wild vs. captive populations (20). Given this, we predicted that neopterin would be elevated in wild compared to captive individuals. Lastly, a common pattern among primates, and humans, is that neopterin changes with age, specifically, it is high in infants, decreases through juvenile and early adulthood, and then increases again with advanced age (18, 21, 23, 44–46). Thus, we predicted a similar pattern in both our wild and captive populations.

## Materials and Methods

### Site Information

We collected *C. imitator* samples from two groups of habituated, white-faced capuchins (26 individuals total) associated with the Capuchinos de Taboga research project. The Capuchinos de Taboga project is located in the Taboga Forest Reserve, a 516-ha tropical dry forest in Northwestern Costa Rica, owned by the Universidad Técnica Nacional. Taboga Forest is subject to extreme anthropogenic pressure but contains some of the highest capuchin densities of any monitored forest fragment in the country. The forest is surrounded by rice and sugarcane farmland that has been intensively cultivated for more than 30 years; approximately 40% of the forest is within 100 m of an anthropogenic edge. The Taboga Forest Reserve is an important piece of a fragmented biological corridor connecting the Tempisque River Basin to the Guanacaste Mountains (47).

We collected *S. apella* samples from five long-term, stable multimale-multifemale social groups and one bachelor male pair (17 individuals total, out of 30 monkeys in the population) at the Language Research Center (LRC) of Georgia State University. This population consists of all subadult and adult monkeys because it is not currently breeding for husbandry purposes (males have been vasectomized). All six groups, including the bachelor pair, live in large, indoor-outdoor enclosures. Capuchins have access to the outdoors at all times unless they have chosen to come inside for testing or during inclement weather. All research at the LRC is non-invasive and consists of cognitive and behavioral testing and non-invasive monitoring, including behavioral observations and endocrinology sampling. Our monkeys are trained to voluntarily enter test boxes affixed to their indoor rooms if they choose to participate in the day's testing; when they do so, we can easily collect clean urine samples from known individuals. Monkeys are fed a diet of fresh fruits and vegetables, supplemented with high protein primate chow several times a day and have *ad libitum* access to running water, including during testing (water sources can be temporarily turned off during urine collection to avoid contaminating urine samples). All participation in testing is voluntary, and monkeys are never deprived of food, water, or access to the outdoors or social partners to motivate testing or sample collection (48).

### Sample Collection

We collected a total of 62 samples from wild *C. imitator* (Jun-Jul 2021). We identified all individuals based on their physical characteristics and ages ranged from 5 weeks to 21 years. Ages were known for all individuals <5 years and estimated (based on body condition) for all individuals >5 years. All individuals that we collected samples from appeared healthy upon visual inspection. We collected urine samples opportunistically between the hours of 5:30 AM and 5:00 PM, primarily using the clean catch method (49) to avoid rainwater, soil, and fecal contamination. We collected samples from leaves only if they did not appear to be contaminated. We transferred urine to 2 ml vials with disposable pipets, immediately stored samples in a portable cooler, and stored them in a −20°C freezer at the end of the field day. We shipped samples via overnight delivery on dry ice to the Core Assay Facility at the University of Michigan and stored them in a −20°C freezer until analysis.

We collected a total of 56 samples from captive *S. apella* (Aug-Nov 2021). We identified all individuals based on their physical characteristics and ages ranged from 9 to 46 years. All individuals that we collected samples from appeared healthy upon visual inspection. We collected samples opportunistically from sterilized, plastic trays placed in individual testing rooms between the hours of 8:00 AM and 2:00 PM. We transferred urine to 15 ml vials with disposable pipets, immediately stored samples in a portable cooler, and transferred them to a −20°C freezer. We then transported samples on ice to the Social Cognition and Primate Behavior Lab at Emory University for analysis.

Out of 118 total samples we included 97 (mean ± SD, 1258.3 ± 1257.2 ng/mL corr. SG) in the analysis. To economize, we only purchased five plates, which means we could only run 105 samples. Therefore, we excluded 13 samples (8 wild, 5 captive) from the initial run, and we were unable to re-run 8 samples (6 wild, 2 captive) that exceeded our 15% intra-sample CVs. We included 48 wild samples (mean ± SD, 2074.4 ± 1358.6 ng/mL corr. SG) and 49 captive samples (458.8 ± 188.5 ng/mL corr. SG) in the analysis. Age range was not evenly distributed across groups (Table 1), wild samples included only one old adult; captive samples included no infant or juvenile samples (because there are no infant or juvenile individuals in the population).

**Table 1:** Sample information for wild and captive individuals.

**Age class**	**Captive (N/n)**	**Wild (N/n)**	**Total (N/n)**	**Mean ±SD (ng/mL corr. SG)**
Infant (0–1yr)	0/0	3/3	3/3	4203.4 ± 1513.0
Juveniles (2–8yr)	0/0	14/29	14/29	1803.6 ± 1237.2
Adults (9–20yr)	9/31	8/15	17/46	990.3 ± 1096.4
Old adults (21–40yr)	8/18	1/1	9/18	609.9 ± 487.3
Total	17/49	26/48	43/97	1258.3 ± 1257.2

*(N/n) Represents (total individuals/total samples). Sampling is uneven across age classes between wild and captive groups. The last column represents average neopterin (ng/mL corr. SG) values for each group*.

### Sample Analysis

We analyzed *C. imitator* samples in the Core Assay Facility at the University of Michigan and *S. apella* samples in the Social Cognition and Primate Behavior Lab at Emory University. We used a commercially available competitive exclusion neopterin ELISA to measure neopterin levels in urine samples (Neopterin ELISA, Ref. RE59321, IBL International GMBH, Hamburg, Germany). The kit was originally manufactured for neopterin detection in human serum, plasma, and urine. We thawed, vortexed, and centrifuged all samples and diluted captive samples to 1:64 and wild samples to 1:128 with manufacturer assay buffer. We ran the assay following the manufacturer's standard protocol. We re-ran or discarded samples with a CV of 15% or higher. Intra-assay variation of wild samples over two plates was 9.8%. Inter-assay variation among the high pool controls was 15.2% and the mid pool control was 2.7%. Intra-assay variation of captive samples over three plates was 7.9%. Inter-assay variation of high pool control was 6.4% and mid pool control was 11.2%. Final neopterin concentration is expressed in μmol/L.

We controlled for sample water volume using both creatinine and specific gravity (SG) to evaluate the suitability of each method for urinary neopterin analysis. We measured creatinine with a commercially available kit using the Jaffe reaction (Creatinine detection kit, Ref. ADI907030A, Enzo Life Sciences Inc., Farmingdale, NY). The kit was originally manufactured for human, mouse, rat, dog, and monkey urine. We thawed, vortexed, and centrifuged all samples and diluted captive samples to 1:8 (if samples ran too high on the standard curve, we adjusted the dilution to 1:16) and wild samples to 1:16 (if samples ran too high on the standard curve, we adjusted dilution to 1:4), with distilled water. We ran the assay using the manufacturer's standard protocol. We re-ran or discarded samples with a CV of 15% or higher. Intra-assay variation of wild samples over 3 plates was 3.3%. Inter-assay variation among the high pool controls was 10.5% and the low pool control was 13.7%. Inter-assay variation of captive samples over two plates was 2.9%. Intra-assay variation of high pool control was 1.8% and low pool control was 0.3%. Final creatinine concentration is expressed in mol/L.

We measured SG using a handheld refractometer (Aichose, Ref. SR0021-ATC) and used methods from Sacco et al. (26) for sample correction. Captive SG mean was 1.0102 and wild SG mean was 1.0195.

### Analytical Validation

We assessed parallelism and accuracy to determine whether the commercial assay effectively measured neopterin concentration in capuchin urine samples. First, we created a wild sample pool and a captive sample pool (representative of all age/sex classes) and serially diluted the pools with manufacturer assay buffer. Given our prediction that wild samples would have higher neopterin concentrations, we adjusted the dilutions accordingly (Wild: 1:16-1024, Captive: 1:4-256). We then ran the serial dilutions and the kit standards on the same plate. Parallelism can be verified both statistically and visually [e.g., (50)]. To assess parallelism, we assigned the diluted pool sample that was closest to 50% binding the concentration of the kit standard that was closest to 50% binding. We then calculated concentrations for the rest of the serial dilution by multiplying up and dividing down the remaining serial dilution by the dilution factor. We plotted the calculated concentrations of the serial dilution against the kit standards and visually inspected whether the slopes were parallel. We also assessed parallelism by examining the interaction between the serial dilution and kit standard using linear modeling. To assess accuracy, we added an aliquot of each kit standard to our captive and wild pool at a 1:1 ratio (i.e., 20 ul of standard: 20 ul of pool). We then calculated the expected concentration of the spiked samples based on the known concentration of the standards and pool. We then compared the observed to expected values.

### Biological Validation

Captive individuals received their rabies booster vaccines (Imrab 3, produced by Merial) during sample collection. We collected post-vaccine samples from five individuals between 0 to 4 days after immunization and compared them to baseline values collected before vaccination or more than 4 days after vaccination. Rabies vaccines are administered by veterinarians every 3 years, suggesting that individuals should have had relatively lower immunity at the time of the booster.

### Statistical Analysis

#### Analytical Analysis

To examine parallelism in our assays we fit multiple linear models (Supplementary Tables 1, 2) using percent binding as the outcome variable and compared them using Akaike information criterion corrected for small sample sizes (AICc). We fit models using the normal distribution, which has been used to establish parallelism in previous publications (50) and the beta distribution, which is more appropriate for data bounded between zero and one (i.e., percent binding). The predictor variables included log neopterin concentration (μmol/L) and type (the categorical variable for pool serial dilution or kit standards). In one model we also included an interaction effect between concentration and type to determine whether the slopes of the two lines (serial dilution and kit standards) differed.

Similarly, we fit multiple linear mixed models (LMM) (Supplementary Table 3) and compared them using AICc to evaluate the relationship between creatinine (μmol/L corr. mol creatinine) and SG-controlled (ng/ml corr. SG) neopterin values. We built two models (1) an intercept model and (2) a model with creatinine controlled neopterin values as the outcome variable predicted by SG controlled neopterin values. In each model, we included a random effect for individual with varying intercepts to control for repeated sampling among individuals. We logged creatinine and SG controlled neopterin values to meet Gaussian model assumptions.

We also evaluated the effect of sex on urinary creatinine (mol/L) to assess the extent to which muscle mass might confound results. We compared two models (Supplementary Table 4) (1) an intercept model and (2) a model with sex as a predictor of urinary creatinine using AICc and included a random effect for individual with varying intercepts to control for repeated sampling among individuals in both models. We logged creatinine values to meet Gaussian model assumptions.

#### Biological Analysis

We assessed changes in urinary neopterin in response to the vaccine by evaluating differences between baseline and post-vaccination samples within individuals. To do this, we calculated the percent difference in means between baseline and post-vaccination samples for each individual. We used z-scores to measure standardized changes between the baseline and the highest post-vaccination value and to determine *p*-values that assessed the significance of these changes. Small sample sizes (*n* = 5) and concerns about overfitting precluded the use of more formal models for these comparisons.

We assessed the effects of environment and age on urinary neopterin using multiple linear mixed models (LMMs) (*n* = 89) and compared them using AICc. Neopterin concentration was the outcome variable for all models but the predictor variables differed among models. Predictors included environment, age, age squared, and an interaction between environment and age (Supplementary Tables 5, 6). All models included a random effect for individual with varying intercepts to control for repeated sampling among individuals. We scaled age in all models for ease of interpretation of the polynomial results.

We built the same models for creatinine-controlled neopterin and SG-controlled neopterin to compare the difference in results between the two methods. We then visually inspected the difference using coefficient plots. Both creatinine-controlled and SG-controlled neopterin values were logged to meet Gaussian model assumptions. We assessed the normality of all model residuals by visually evaluating a histogram and Q-Q plot of the residuals and confirmed assumptions were met for both models. We conducted all analyses in R 4.1.3 (51). All data and code necessary to replicate our results and produce our figures are available at https://github.com/andrewjohnmarshall/neopterin_validation.

## Results

### Analytical Validation

**Figure 1:**
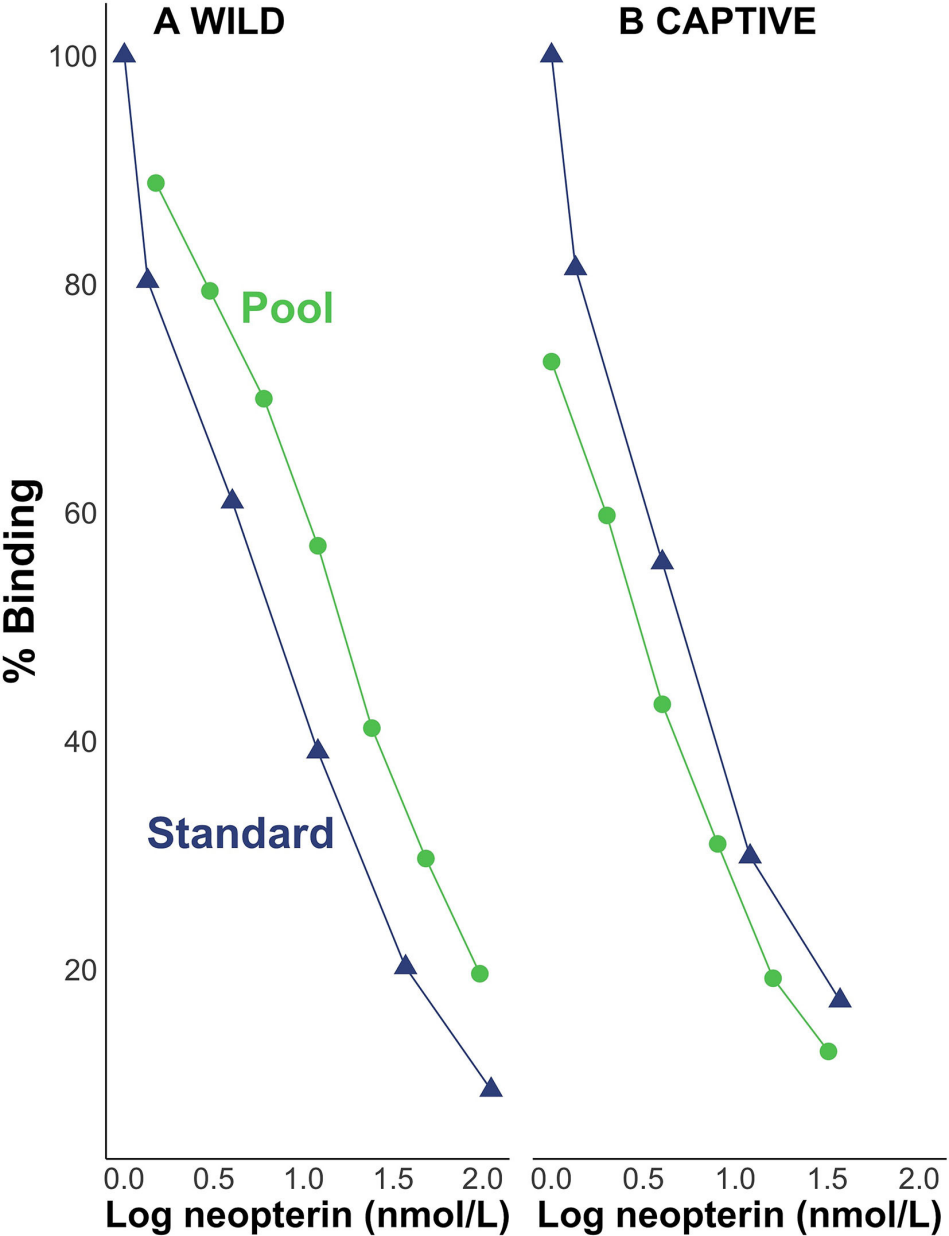
Percent binding of pool serial dilutions of **(A)** wild and **(B)** captive individuals in relation to the standard curve.

Visual inspection suggests that pool serial dilutions were parallel to the standard curve in the binding range of 20–80% for both wild (Figure 1A) and captive (Figure 1B) individuals. We found the beta distribution fit better for the intercept model; but when we included the predictor variables, the two top models were based on the normal distribution. For the wild samples, the best-fitting model (91% of the model weight, ω) did not include an interaction effect between log neopterin concentration (μmol/L) and type (the categorical variable for pool serial dilution or kit standards) indicating that the slopes did not differ meaningfully by type. The next best-fitting model (ω = 8%) included the interaction effect but showed the interaction between concentration and type is not a reliable predictor of percent binding ($\beta_{\text{concentratio}{\text{n}}^{*}type}$ = −0.0003, SE = 0.0004). For the captive samples, the best-fitting model (ω = 76%) did not include an interaction effect between log neopterin concentration and type indicating that the slopes did not differ meaningfully by type. The next best-fitting model (ω = 10%) included the interaction effect but showed the interaction between concentration and type is not a reliable predictor of percent binding ($\beta_{\text{concentratio}{\text{n}}^{*}type}$ = −0.07, SE = 0.06).

For evaluation of accuracy, the average recovery for spiked wild samples was 98.8 (range 86.8 – 109.5) and spiked captive samples was 109.0 (range 102.23 – 123.9) (Table 2).

**Table 2:** Accuracy of urinary neopterin of pools in captive and wild individuals.

**Pool**	**Standard (nmol/L)**	**Expected (nmol/L)**	**Measured (nmol/L)**	**Recovery (%)**
**Captive**	0.0	2.6	3.3	123.9
	0.7	3.3	3.4	102.5
	2.0	4.6	5.4	116.4
	6.0	8.6	9.0	104.6
	18.5	21.1	22.1	104.3
	55.0	58.1	59.6	102.5
				Mean = 109.0
**Wild**	0.0	6.3	5.4	86.8
	0.7	10.0	6.4	92.3
	2.0	8.3	8.0	96.6
	6.0	12.3	12.6	102.9
	18.5	24.8	26.0	104.9
	55.0	61.8	67.6	109.5
				Mean = 98.8

*The pool (captive = 1:64 dilution, wild = 1:128) was spiked with 5 neopterin concentrations (kit standards)*.

Creatinine-controlled values were tightly correlated with specific gravity (SG)-controlled values. The model of creatinine and SG-controlled values performed better (ω = 100%) than the intercept model for creatinine-controlled values (ω = 0%) and SG-controlled values were a meaningful predictor of creatinine-controlled values (β_SGcontrolled_ = 2.0, SE = 1.05).

Urinary creatine values did not differ meaningfully between females and males. The intercept model of urinary creatinine (ω = 79%) performed better than the model that included the sex predictor (ω = 21%) and sex was not a reliable predictor of creatinine (β_sex_ = 1.19, SE = 1.29).

### Biological Validation: Vaccine Response

**Figure 2:**
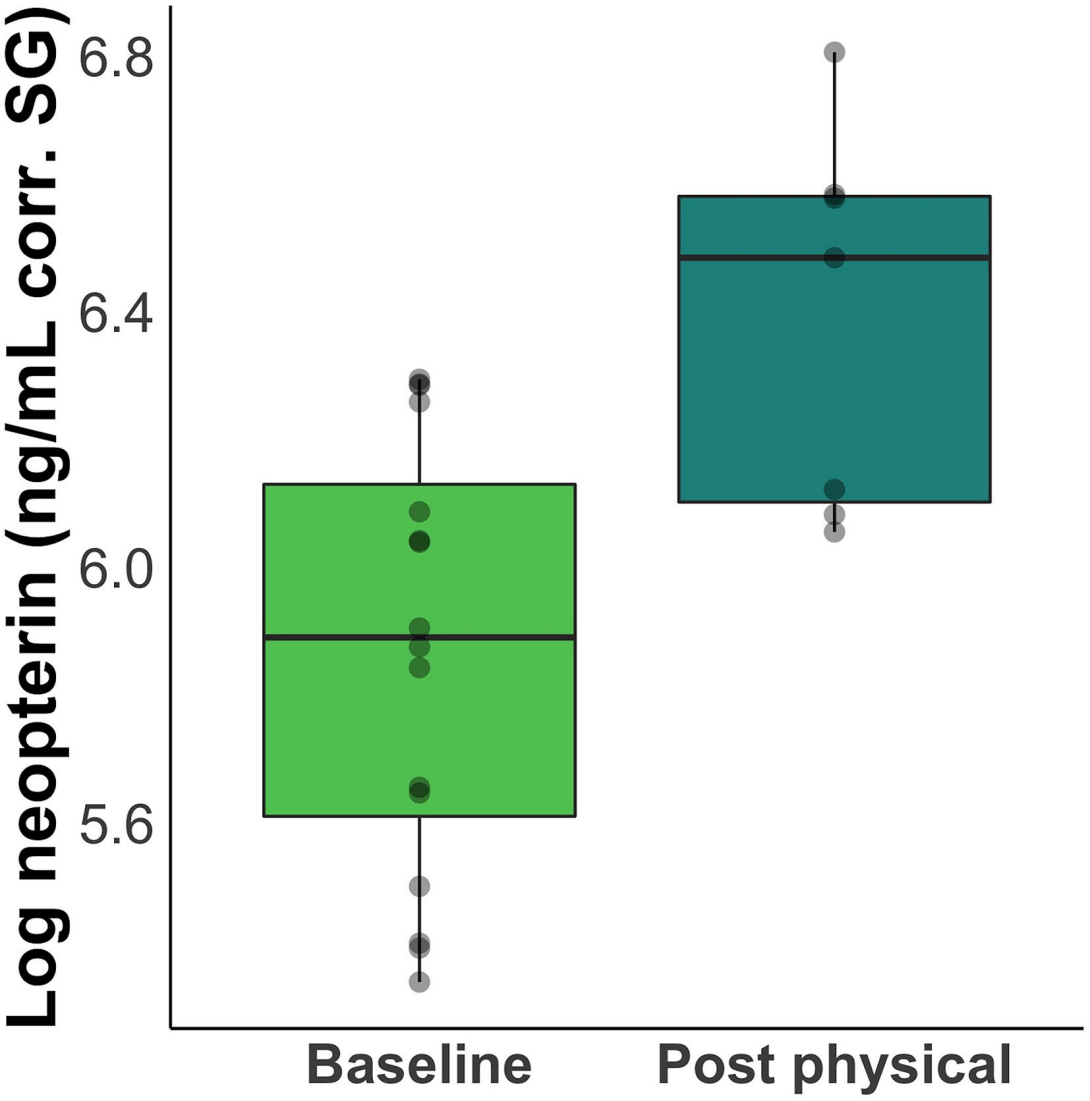
Difference between baseline and post-inoculation neopterin concentrations. Boxplots show a 66% increase in mean neopterin concentration 1 – 4 days after inoculation.

**Figure 3:**
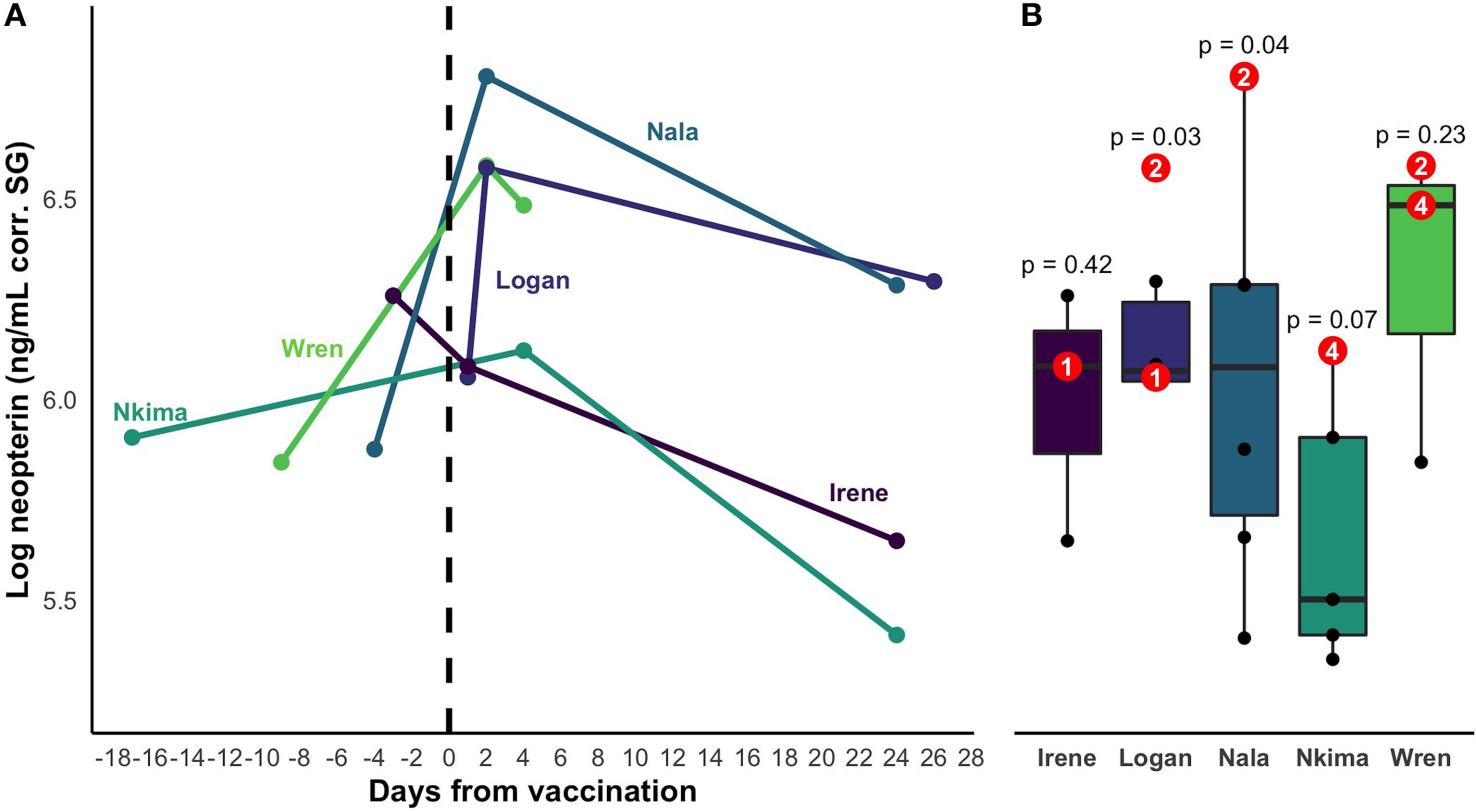
**(A)** Change in neopterin concentration over time for vaccinated individuals. The timeline encompasses the closest samples collected before and after vaccination for each individual. Day zero represents the day of vaccination. Colors represent distinct individual animals. **(B)** Range of baseline values of the 5 vaccinated individuals. Post-vaccination values are shown in red. Superimposed numbers on red values represent the number of days a sample was collected after vaccination. *P*-values represent the statistical significance of the difference between an individual's highest post-vaccination concentration and the range of their other neopterin values.

Neopterin concentration increased after rabies booster vaccination in four out of five individuals (mean percent increase across all five subjects = 66%; Figure 2). Peak increase occurred 2 days after vaccination, but neopterin remained elevated until 4 days after vaccination compared to baseline levels (Figure 3A). Neopterin increase post-vaccination is statistically significant in two individuals only (*p* = 0.03 and 0.04), although all individuals except Irene showed increases in neopterin levels post-vaccination (Figures 3A,B).

### Model Results for Age and Environment

**Figure 4:**
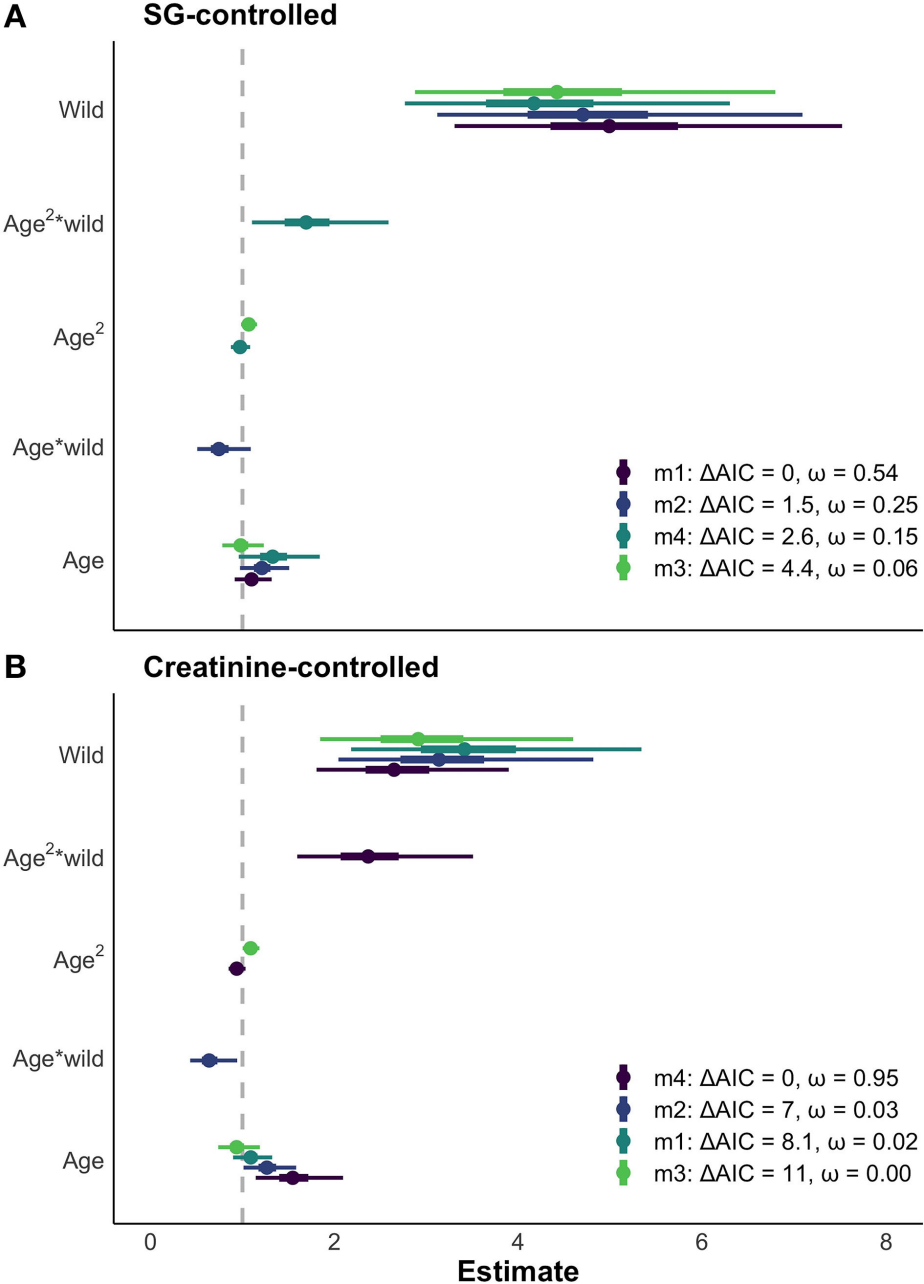
**(A)** Comparison of models with > 3% of model weight for SG controlled neopterin values. Similar results of age and environment are seen across all models. Thick bars represent 50% CI, thin bars represent 95% CI. **(B)** The same four models fitted with creatinine-controlled values. The SG and creatinine controlled models show similar results for the effect of age and environment on neopterin.

**Figure 5:**
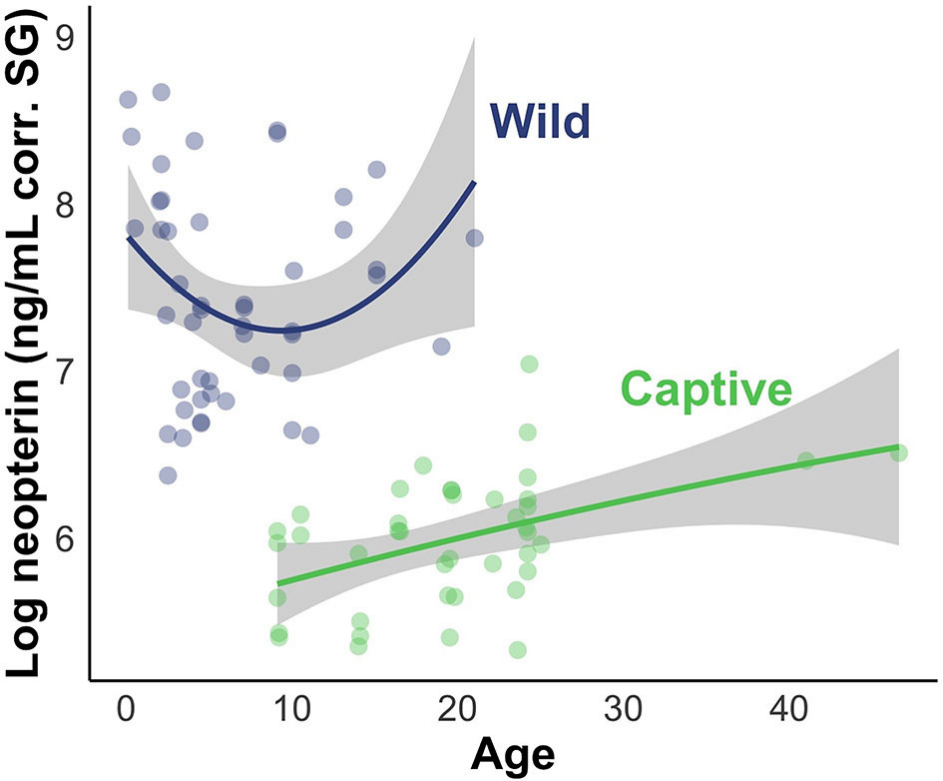
Visual representation of LMM evaluating the effect of subject age and environment on neopterin values. Shading represents 95% CI.

The results of the LMMs were consistent across models (Figure 4) and all models held a meaningful portion of the model weight (ω > 3%). We saw minimal differences between the creatinine-controlled and SG-controlled models (Figure 4). Given that the values showed a tight correlation in the above analysis, we report the results of the SG-controlled model only. Since all of the SG-controlled LMMs held a substantial portion of model weight, we averaged the models for ease of interpretation and discussion. Environment was a reliable predictor of neopterin concentration (β_wild_ = 4.75, SE = 1.25); the wild population had substantially higher neopterin than the captive population (Figures 4, 5). Neopterin showed a modest increase with age (β_age_ = 1.15, SE = 1.15) but we found a very small interaction effect between age and environment ($\beta_{\text{ag}{\text{e}}^{*}wild}$ = 0.74, SE = 1.22). We also found no effect for polynomial age ($\beta_{\text{ag}{\text{e}}^{2}}$ = 0.99, SE = 1.00) but we found a modest effect of the interaction between polynomial age and environment ($\beta_{\text{ag}{\text{e}}^{2*}wild}$ = 1.69, SE = 1.25) showing that the wild population had a modest convex trend in neopterin with age. Neopterin was elevated in early and late-life (Figure 5).

## Discussion

In this study, we validated methods for collection and analysis of urinary neopterin in capuchin monkeys. In line with our predictions, we found that neopterin increased after immune perturbation (rabies vaccine booster), varied by environmental condition, and mirrored expected trends in immune system ontogeny. Our results contribute to a growing number of studies examining urinary neopterin as a biomarker of health in wild primates, which to date have focused almost exclusively on catarrhine primates. To our knowledge, this study presents the first complete validation of urinary neopterin in a platyrrhine species, highlighting both the effectiveness of commercially available assay kits in measuring neopterin in capuchin urine and the biological validity of using urinary neopterin as a health monitoring tool in this taxon.

Results from this study support previous findings that urinary neopterin can be measured in platyrrhine urine. Previous studies have found that urinary neopterin is detectable via commercially available assays in three platyrrhine families (26). Our study adds to these findings in three key ways. First, it provides a complete analytical validation for how urinary neopterin performs in platyrrhine urine. While detection is a first step, there are two key components necessary to assess the validity of an enzyme immunoassay method: parallelism and accuracy (50, 52). We found that, for capuchins, these kits perform well on both counts.

Second, we systematically compared two commonly used methods that control for dilution of urine in neopterin analyses. In line with previous research, we found that both creatinine and specific gravity (SG) were tightly correlated (36, 37) suggesting that either can be used to standardize urinary neopterin. Some wild primate urinary neopterin studies used SG to control for urine dilution, most likely because it is a more field-friendly and economical method to control for sample concentration than creatinine assays (18, 19, 21–24, 26). We now know SG is a sufficient method to control for urine dilution for neopterin analysis in capuchin monkeys. We also found no meaningful difference in creatinine levels between the sexes. Given these findings, both creatinine and SG are suitable methods to control for urine concentration in capuchins. We recommend using SG because it is a more field-friendly and economical method to control for sample concentration than creatinine assays.

Third, this study presents the first biological neopterin validation in wild and captive capuchin species. In our captive population, we saw an average 66% increase (Figure 2) in neopterin concentration 1 – 4 days after inoculation with a rabies booster vaccine (Figure 3). Inoculation research in children has shown a similar neopterin response to vaccination with neopterin increasing following inoculation with a live measles-mumps vaccine (41). An important distinction here is that children exhibit an increase in neopterin after initial vaccination but not following a booster shot. For the capuchins, the rabies vaccine was a booster, thus the following increase in neopterin levels could be a result of the adjuvant in the IMRAB 3 rabies vaccine, meant to stimulate an immune response. Additionally, the increase in neopterin levels could suggest immunity was waning in this population at the time of vaccination, causing a pronounced immune response. It is possible the individual who did not exhibit an immune response had a higher level of residual rabies immunity. More research needs to be done, however, to fully understand the relationship between neopterin and the immune response in capuchins (e.g., the effects of inflammatory response and disease on neopterin concentrations).

Previous studies have found a large degree of variability in baseline neopterin in humans (53) and primates [barbary macaques, (24); chimpanzees, (18, 19, 21, 22); bonobos, (23); emperor tamarins, (26); saddle-back tamarins, (26)], making it difficult to determine “normal”, baseline neopterin values for primate species. Importantly, there is overlap between baseline neopterin and neopterin after immune activation. This pattern was seen in chimpanzees with respiratory infections (18, 19) and humans with tuberculosis (53), making it difficult to determine baseline neopterin vs. neopterin post innate immune activation without within-subject comparison. Our values show the same trend; baseline neopterin is variable among individuals and some individuals' baseline values overlap the post-vaccination values of others (Figure 3). We do not expect diurnal variation to cause differences in neopterin values because neopterin does not vary diurnally in bonobos (18) and it is excreted in surprisingly constant proportions (11). Given the extreme variation in neopterin among individuals, it is currently only appropriate to conduct within-subject comparison when measuring innate immune response. Innate immune activation may be individual-specific, thus, neopterin may only be effectively used for longitudinal individual sampling. However, it is also possible that the variation within and between individuals could be a result of underlying conditions or co-infections (26). Future studies should employ individual longitudinal sampling to evaluate expected individual variation and begin to answer these questions. In particular, an understanding of expected individual variation could help control for undiagnosed underlying conditions/coinfections.

Our data show that wild capuchins have significantly higher neopterin levels than captive capuchins. High neopterin in wild populations suggests higher disease burdens and the need to invest more in the innate immune system (20). In contrast, captive animals live in much more sterilized environments with strict protocols for food and habitat sanitation, no outside source for conspecific disease transmission, access to preventative medicine [e.g., parasite treatment, immunizations; (54)], and pest control to limit vector-borne disease (55). Similarly, humans (56), birds (42), and dolphins (43) show upregulation of the immune system in environments with suspected higher pathogen load.

The environmental variables driving differences in neopterin are probably not as simple as our captive vs. wild comparison implies. Notably, our wild population lives in an area of high anthropogenic disturbance (47) and animals in anthropogenic environments are often subject to increased risk of zoonotic disease and generally higher pathogen exposure (31, 33, 57). Difference in neopterin between wild and captive individuals may depend on degree of anthropogenic degradation in the wild environment. Individuals living in more “pristine” environments [i.e., bonobos; (23)] may show little difference in immune activation from captive individuals because of the shared evolutionary history with common pathogens in their environment (23). Further, glucocorticoids are known to affect immune system activation (58) and may differ between wild and captive populations. However, no research, to our knowledge, has investigated the connection between glucocorticoids and neopterin. More research is needed on this kind of environmental comparison to evaluate trends in innate immune activation in response to environmental factors.

While there is little data providing strong comparisons between closely related species, given that the immune system is highly conserved across vertebrates (59, 60), we believe that our results reflect differences in innate immune system activation as a result of environmental conditions, rather than taxonomic differences between *Sapajus* and *Cebus*. We note, however, that comparison between different genera is a limitation in our study and one that needs to be further addressed with a comparison on a wild *Sapajus* or captive *Cebus* population.

Given the previous research on neopterin age trend, we predicted that neopterin would be highest in early and late life. Our predictions were largely met, however, the convex age trend was only observed in the wild population because the captive population did not include infants and juveniles (Table 1). The captive population showed a neopterin increase with advanced age indicating a convex trend would likely have been observed in the captive population were we to have had samples from infants and juveniles. This result is similar to other primate studies that excluded infants and juveniles (24). Future studies should focus on sampling a wide range of age classes to further investigate the effects of age.

The wild population shows a larger neopterin increase in mid-late adulthood compared to the captive population (Figure 5). Life history patterns differ between wild and captive populations; captive individuals often have earlier age of menarche, first birth, and shorter interbirth intervals (61), probably due to relaxed environmental constraints (e.g., food availability, disease). Neopterin increase with immunosenescence may show similar differences between wild and captive populations in that the captive immune system senesces at a later age with decreased intensity. Further comparative work is needed to understand how immunosenescence differs between wild and captive populations. Neopterin has potential as a marker of immune system ontogeny because it mirrors expected change in immune system function with age.

Our research contributes to the growing number of studies integrating neopterin as a valuable maker of health and immune status for wild primates. Specifically, we add an important comparative data set, examining neopterin and immune response in platyrrhine primates, that has been largely missing from neopterin studies. It is our goal that the methods and findings of this study spur more research on neopterin as a non-invasive measure of health and longevity in platyrrhines. An important future trajectory is using neopterin as a tool to study the effects of anthropogenic perturbation on primates' health, a necessity since primates are increasingly utilizing anthropogenic environments due to human encroachment (30). Captive facilities may also find neopterin useful as a relatively quick and non-invasive measure of health in captive individuals, for instance, to be used to track at-risk animals non-invasively.

## Data Availability Statement

The datasets presented in this study can be found in online repositories. The names of the repository/repositories and accession number(s) can be found below: https://github.com/andrewjohnmarshall/neopterin_validation.

## Ethics Statement

All protocols involved in this study were previously approved by Emory University IACUC (#PROTO202100033) and Georgia State University IACUC (#A20018). Additionally, all protocols and procedures used in this study complied with the relevant legal requirements governing animal research in Costa Rica and in the United States of America.

## Author Contributions

JL, MB, and AM: conception and design and drafting of the manuscript. JL, MB, and SB: sample acquisition. JL and MB: sample analysis. JL and AM: statistical analysis. JL: R code and figures. All authors reviewed and approved the final version of the manuscript.

## Funding

Wild sample collection was funded by the University of Michigan Department of Anthropology and the University of Michigan International Institute Latin American and Caribbean Tinker Field Grant. Captive sample collection and sample analysis was funded by Emory University.

## Conflict of Interest

The authors declare that the research was conducted in the absence of any commercial or financial relationships that could be construed as a potential conflict of interest.

## Publisher's Note

All claims expressed in this article are solely those of the authors and do not necessarily represent those of their affiliated organizations, or those of the publisher, the editors and the reviewers. Any product that may be evaluated in this article, or claim that may be made by its manufacturer, is not guaranteed or endorsed by the publisher.

## References

[1] Freeland WJ. Pathogens and the evolution of primate sociality. Biotropica. (1976) 8:12–24. 10.2307/2387816

[2] Crofoot MC Norton TM Lessnau RG Viner TC Chen TC Mazzaro LM . Field anesthesia and health assessment of free-ranging Cebus capucinus in Panama. Int J Primatol. (2009) 30:125–41. 10.1007/s10764-009-9333-6

[3] Soto-Calderón ID Acevedo-Garcés YA Álvarez-Cardona J Hernández-Castro C García-Montoya GM. Physiological and parasitological implications of living in a city: the case of the white-footed tamarin (Saguinus leucopus). Am J Primatol. (2016) 78:1272–81. 10.1002/ajp.22581 27404890

[4] Teixeira MG Ferreira AF Colaço AA Ferreira SF de Melo Benvenutti ME Queiroga FLPG. Hematologic and blood chemistry values of healthy Cebus flavius kept in northeast of Brazil. J Med Primatol. (2013) 42:51–6. 10.1111/jmp.12036 23350904

[5] Nunn CL. Primate disease ecology in comparative and theoretical perspective. Am J Primatol. (2012) 74:497–509. 10.1002/ajp.21986 22539269

[6] Leendertz SAJ Metzger S Skjerve E Deschner T Boesch C Riedel J . A longitudinal study of urinary dipstick parameters in wild chimpanzees (Pan troglodytes verus) in Côte d'Ivoire. Am J Primatol. (2010) 72:689–98. 10.1002/ajp.20825 20333735

[7] Archie EA Altmann J Alberts SC. Social status predicts wound healing in wild baboons. Proc Nat Acad Sci. (2012) 109:9017–22. 10.1073/pnas.1206391109 22615389 PMC3384186

[8] Actor JK. A functional overview of the immune system and immune components. in Introductory Immunology, eds. Actor J.K.. Academic Press. (2019) p. 1–16. 10.1016/B978-0-12-816572-0.00001-2

[9] McDade TW Georgiev AV Kuzawa CW. Trade-offs between acquired and innate immune defenses in humans. Evol Med Public Health. (2016) 2016:1–16. 10.1093/emph/eov033 26739325 PMC4703052

[10] Hamerlinck FFV. Neopterin: a review. Exp Dermatol. (1999) 8:167–76. 10.1111/j.1600-0625.1999.tb00367.x 10389633

[11] Murr C Widner B Wirleitner B Fuchs D. Neopterin as a marker for immune system activation. Curr Drug Metab. (2002) 3:175–87. 10.2174/1389200024605082 12003349

[12] Widner B Murr C Wirleitner B Mayr C Spöttl N Baier-Bitterlich G . The importance of neopterin as a laboratory diagnostic marker of immune activation. Pteridines. (1999) 10:101–11. 10.1515/pteridines.1999.10.3.10128712531

[13] Wirleitner B Reider D Ebner S Böck G Widner B Jaeger M . Monocyte-derived dendritic cells release neopterin. J Leukoc Biol. (2002) 72:1148–53. 10.1189/jlb.72.6.1148 12488496

[14] Fuchs D Hausen A Reibnegger G Werner ER Dierich MP. Neopterin as a marker for activated cell-mediated immunity: application in HIV infection. Immunol Today. (1988) 9:150–5. 10.1016/0167-5699(88)91203-0 3076770

[15] Fendrich C Lüke W Stahl-Hennig C Herchenröder O Fuchs D Wachter H . Urinary neopterin concentrations in rhesus monkeys after infection with simian immunodeficiency virus (SIVmac 251). AIDS. (1989) 3:305–7. 10.1097/00002030-198905000-00010 2548536

[16] Stahl-Hennig C Fendrich C Lüke W Widner B Hunsmann G Fuchs D. Urinary neopterin indicates early infection and disease progression: Model studies with simian and human immunodeficiency viruses in macaques. Pteridines. (2002) 13:1–8. 10.1515/pteridines.2002.13.1.1

[17] Higham JP Kraus C Stahl-Hennig C Engelhardt A Fuchs D Heistermann M. Evaluating noninvasive markers of nonhuman primate immune activation and inflammation. Am J Phys Anthropol. (2015) 158:673–84. 10.1002/ajpa.22821 26250063

[18] Behringer V Stevens JM Leendertz FH Hohmann G Deschner T. Validation of a method for the assessment of urinary neopterin levels to monitor health status in non-human-primate species. Front Physiol. (2017) 8:51. 10.3389/fphys.2017.00051 28220080 PMC5292569

[19] Wu DF Behringer V Wittig RM Leendertz FH Deschner T. Urinary neopterin levels increase and predict survival during a respiratory outbreak in wild chimpanzees (Taï National Park, Côte d'Ivoire). Sci Rep. (2018) 8:1–9. 10.1038/s41598-018-31563-7 30190614 PMC6127264

[20] Behringer V Stevens JM Wittig RM Crockford C Zuberbühler K Leendertz FH . Elevated neopterin levels in wild, healthy chimpanzees indicate constant investment in unspecific immune system. BMC Zoology. (2019) 4:1–7. 10.1186/s40850-019-0041-1

[21] Negrey JD Behringer V Langergraber KE Deschner T. Urinary neopterin of wild chimpanzees indicates that cell-mediated immune activity varies by age, sex, and female reproductive status. Sci Rep. (2021) 11:1–11. 10.1038/s41598-021-88401-6 33927233 PMC8085242

[22] Löhrich T Behringer V Wittig RM Deschner T Leendertz FH. The use of neopterin as a noninvasive marker in monitoring diseases in wild chimpanzees. Ecohealth. (2018) 15:792–803. 10.1007/s10393-018-1357-y 30117002

[23] Behringer V Deimel C Stevens JM Kreyer M Lee SM Hohmann G . Cell-mediated immune ontogeny is affected by sex but not environmental context in a long-lived primate species. Front Ecol Evol. (2021) 9:272. 10.3389/fevo.2021.629094

[24] Müller N Heistermann M Strube C Schülke O Ostner J. Age, but not anthelmintic treatment, is associated with urinary neopterin levels in semi-free ranging Barbary macaques. Sci Rep. (2017) 7:1–11. 10.1038/srep41973 28155915 PMC5290464

[25] IUCN. The IUCN Red List of Threatened Species. Version 2021-X. (2021). Available online at: https://www.iucnredlist.org (accessed March 6, 2022).

[26] Sacco AJ Mayhew JA Watsa M Erkenswick G Binder AK. Detection of neopterin in the urine of captive and wild platyrrhines. BMC Zoology. (2020) 5:1–8. 10.1186/s40850-020-00051-9

[27] Lahoz M Kauffman MA Carfagnini J Vidal A Papouchado M Sterin-Prync A . Pharmacokinetics and pharmacodynamics of interferon beta 1a in Cebus apella. J Med Primatol. (2009) 38:187–91. 10.1111/j.1600-0684.2008.00333.x 19054274

[28] Jack K. The Cebines. in Primates in Perspective. ed. Campbell C.J. Fuentes A. Mackinnon K. Bearder S.K. Stumpf R.B.. New York: Oxford University Press. (2007). p. 107–122.

[29] Lynch Alfaro JW Izar P Ferreira RG. Capuchin monkey research priorities and urgent issues. Am J Primatol. (2014) 76:705–20. 10.1002/ajp.22269 24668460

[30] Estrada A Garber PA Rylands AB Roos C Fernandez-Duque E Di Fiore A . Impending extinction crisis of the world's primates: Why primates matter. Science advances. (2017) 3:e1600946. 10.1126/sciadv.1600946 28116351 PMC5242557

[31] Chapman CA Gillespie TR Goldberg TL. Primates and the ecology of their infectious diseases: how will anthropogenic change affect host-parasite interactions? Evolution Anthropol. (2005) 14:134–44. 10.1002/evan.20068

[32] Deem SL Karesh WB Weisman W. Putting theory into practice: wildlife health in conservation. Conserv Biol. (2001) 15:1224–33. 10.1046/j.1523-1739.2001.00336.x

[33] Daszak P Cunningham AA Hyatt AD. Emerging infectious diseases of wildlife–threats to biodiversity and human health. Science. (2000) 287:443–9. 10.1126/science.287.5452.443 10642539

[34] Benítez ME Brosnan SF Fragaszy DM. Behavioral biology of capuchin monkeys. in Behavioral Biology of Laboratory Animals, ed Coleman C.K. Schaprio S.J.. Boca Raton: CRC Press. (2021). p. 421–436. 10.1201/9780429019517-29

[35] Heistermann M Higham JP. Urinary neopterin, a non-invasive marker of mammalian cellular immune activation, is highly stable under field conditions. Sci Rep. (2015) 5:1–13. 10.1038/srep16308 26549509 PMC4637859

[36] Anestis SF Breakey AA Beuerlein MM Bribiescas RG. Specific gravity as an alternative to creatinine for estimating urine concentration in captive and wild chimpanzee (Pan troglodytes) samples. Am J Primatol. (2009) 71:130–5. 10.1002/ajp.20631 18973242

[37] Miller RC Brindle E Holman DJ Shofer J Klein NA Soules MR . Comparison of specific gravity and creatinine for normalizing urinary reproductive hormone concentrations. Clin Chem. (2004) 50:924–32. 10.1373/clinchem.2004.032292 15105350

[38] Heymsfield SB Arteaga C McManus C Smith J Moffitt S. Measurement of muscle mass in humans: validity of the 24-hour urinary creatinine method. Am J Clin Nutr. (1983) 37:478–94. 10.1093/ajcn/37.3.478 6829490

[39] Emery Thompson M Muller MN Wrangham RW. Variation in muscle mass in wild chimpanzees: Application of a modified urinary creatinine method. Am J Phys Anthropol. (2012) 149:622–7. 10.1002/ajpa.22157 23077085

[40] Berlin A Alessio L Sesana G Dell'Orto A Ghezzi I. Problems concerning the usefulness of adjustment of urinary cadmium for creatinine and specific gravity. Int Arch Occup Environ Health. (1985) 55:107–111. 10.1007/BF00378372 3988354

[41] Fuchs D Hausen A Reibnegger G Werner ER Werner-Felmayer G Wachter H. Distinct neopterin excretion patterns after vaccination. Pteridines. (1990) 2:147–9. 10.1515/pteridines.1990.2.3.147

[42] Buehler DM Piersma T Irene Tieleman B. Captive and free-living red knots Calidris canutus exhibit differences in non-induced immunity that suggest different immune strategies in different environments. J Avian Biol. (2008) 39:560–6. 10.1111/j.0908-8857.2008.04408.x

[43] Fair PA Schaefer AM Houser DS Bossart GD Romano TA Champagne CD . The environment as a driver of immune and endocrine responses in dolphins (Tursiops truncatus). PLoS ONE. (2017) 12:e0176202. 10.1371/journal.pone.0176202 28467830 PMC5415355

[44] Dibakou SE Souza A Boundenga L Givalois L Mercier-Delarue S Simon F . Ecological, parasitological and individual determinants of plasma neopterin levels in a natural mandrill population. Int J Parasitol. (2020) 11:198–206. 10.1016/j.ijppaw.2020.02.009 32140406 PMC7049574

[45] Hearps AC Martin GE Angelovich TA Cheng WJ Maisa A Landay AL . Aging is associated with chronic innate immune activation and dysregulation of monocyte phenotype and function. Aging Cell. (2012) 11:867–75. 10.1111/j.1474-9726.2012.00851.x 22708967

[46] Winkler C Frick B Schroecksnadel K Wirleitner B Fuchs D. Follow-up of urinary neopterin concentrations in two healthy children until adolescence. Pteridines. (2003) 14:102–7. 10.1515/pteridines.2003.14.3.102

[47] Tinsley Johnson E Benítez ME Fuentes A McLean CR Norford AB Ordoñez JC . High density of white-faced capuchins (Cebus capucinus) and habitat quality in the Taboga Forest of Costa Rica. Am J Primatol. (2020) 82:e23096. 10.1002/ajp.23096 31976575

[48] Benítez ME Sosnowski MJ Tomeo OB Brosnan SF. Urinary oxytocin in capuchin monkeys: Validation and the influence of social behavior. Am J Primatol. (2018) 80:e22877. 10.1002/ajp.22877 29797338

[49] Bergstrom ML Emery Thompson M Melin AD Fedigan LM. Using urinary parameters to estimate seasonal variation in the physical condition of female white-faced capuchin monkeys (Cebus capucinus imitator). Am J Phys Anthropol. (2017) 163:707–15. 10.1002/ajpa.23239 28555757

[50] Beehner J Alfaro JF Allen C Benítez ME Bergman TJ Buehler MS . Steroid hormone validations in wild capuchins at an on-site laboratory. Gen Comp Endocrinol. (In Press). 10.1016/j.ygcen.2022.114109 36007549

[51] R Core Team,. R: A language environment for statistical computing. R Foundation for Statistical Computing, Vienna, Austria. (2021). Available online at: https://www.R-project.org/ (accessed June 15, 2022).

[52] Andreasson U Perret-Liaudet A van Waalwijk van Doorn LJ Blennow K Chiasserini D Engelborghs S . A practical guide to immunoassay method validation. Front Neurol. (2015) 6:179. 10.3389/fneur.2015.00179 26347708 PMC4541289

[53] Eisenhut M Hargreaves DS Scott A Housley D Walters A Mulla R. Determination of urinary neopterin/creatinine ratio to distinguish active tuberculosis from latent Mycobacterium tuberculosis infection. J Biomark. (2016) 2016:1–6. 10.1155/2016/5643853 27433370 PMC4940561

[54] Backues K Clyde V Denver M Fiorello C Hilsenroth R Lamberski N . Guidelines for zoo and aquarium veterinary medical programs and veterinary hospitals. J Zoo Wildlife Med. (2011) 42:176–92. Available online at: https://www.jstor.org/stable/41262606 22946394

[55] Mackie RA. The control of pests in the san diego zoo. J Environ Health. (1967) 29:329–39.

[56] Solomons NW Mazariegos M Brown KH Klasing K. The underprivileged, developing country child: environmental contamination and growth failure revisited. Nutr Rev. (1993) 51:327–32. 10.1111/j.1753-4887.1993.tb03758.x 8108032

[57] Gillespie TR Chapman CA Greiner EC. Effects of logging on gastrointestinal parasite infections and infection risk in African primates. J Appl Ecol. (2005) 42:699–707. 10.1111/j.1365-2664.2005.01049.x

[58] Glaser R Kiecolt-Glaser JK. Stress-induced immune dysfunction: implications for health. Nat Rev Immunol. (2005) 5:243–51. 10.1038/nri1571 15738954

[59] Ausubel FM. Are innate immune signaling pathways in plants and animals conserved? Nat Immunol. (2005) 6:973–979. 10.1038/ni1253 16177805

[60] Medzhitov R Janeway CA. An ancient system of host defense. Curr Opin Immunol. (1998) 10:12–5. 10.1016/S0952-7915(98)80024-1 9523104

[61] Atsalis S Videan E. Reproductive aging in captive and wild common chimpanzees: factors influencing the rate of follicular depletion. Am J Primatol. (2009) 71:271–82. 10.1002/ajp.20650 19067363

