# Supplementary material for: Validating Urinary Neopterin as a Biomarker of Immune Response in Captive and Wild Capuchin Monkeys
Source: Front Vet Sci. 2022 Jul 13;9:918036. doi: 10.3389/fvets.2022.918036 (PMC9326447; doi:10.3389/fvets.2022.918036)
Supplement: Supplementary file 1 [file Data_Sheet_1.PDF]

Supplementary Material

1 Supplementary Figures and Tables

1.1 Supplementary Figures

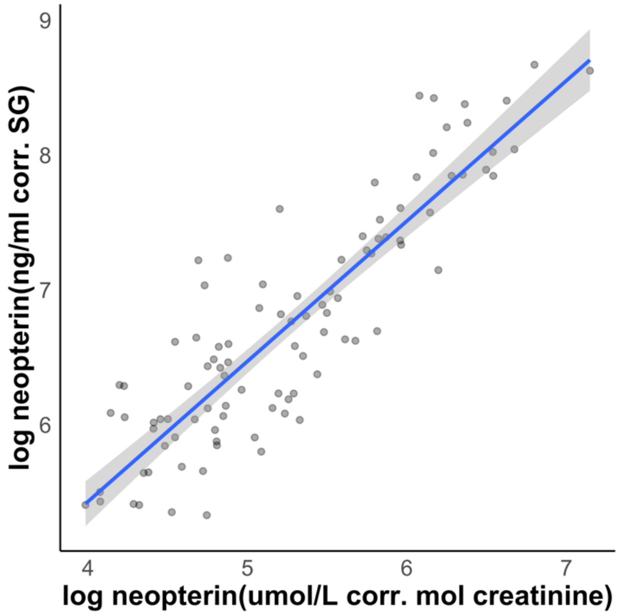

Supplementary Figure 1: Linear regression of log SG controlled neopterin and log creatinine controlled neopterin. Values show a tight correlation (Pearson's correlation = 0.89). The shaded area represents 95% CI.

1.2 Supplementary Tables

Supplementary Table 1: Full list of four models run on wild validation dataset (n = 13), models ordered by ΔAICc.

Model	Model distribution	Model description	Model syntax (%binding ~)	df	ΔAICc	AICc weight
m2.norm	normal	log concentration + type	~ log_conc + type	4	0	0.92
m1. norm	normal	log concentration * type	~ log_conc * type	5	5	0.08
m2. beta	beta	log concentration + type	~ log_conc + type	4	9.2	< 0.01
m1.beta	beta	log concentration * type	~ log_conc * type	5	9.6	< 0.01

Supplementary Table 2: Full list of four models run on captive validation dataset (n = 13), models ordered by ΔAICc.

Model	Model distribution	Model description	Model syntax (%binding ~)	df	ΔAICc	AICc weight
m2.norm	normal	log concentration + type	~ log_conc + type	4	0	0.76
m1. norm	normal	log concentration * type	~ log_conc * type	5	4	0.1
m2. beta	beta	log concentration + type	~ log_conc + type	4	4.4	0.08
m1.beta	beta	log concentration * type	~ log_conc * type	5	5.2	0.06

Supplementary Table 3: Full list of two models run on creatinine-controlled versus specifics gravity (SG)-controlled neopterin values (n = 97), models ordered by ΔAICc.

Model	Model description	Model syntax (log creatinine-controlled neopterin ~)	df	ΔAICc	AICc weight
m2	log SG controlled neopterin + ID (random effect)	~ logneo_sg + (1|ID)	2	0	1
m1.intercept	intercept + ID (random effect)	~ 1 + (1|ID)	88	3	<0.01

Supplementary Table 4: Full list of two models run on urinary creatinine values (n = 97), models ordered by ΔAICc.

Model	Model description	Model syntax (log urinary creatinine ~)	df	ΔAICc	AICc weight
m1.intercept	intercept + ID (random effect)	~ 1 + (1|ID)	0	3	0.79
m2	sex + ID (random effect)	~ sex + (1|ID)	2.6	4	0.21

Supplementary Table 5: Full list of four models run on SG-controlled neopterin values (n = 89), models ordered by ΔAICc.

Model	Model description	Model syntax (log SG-controlled neopterin ~)	df	ΔAICc	AICc weight
m1	age + environment + ID (random effect)	~ scale_age + type + (1|ID)	5	0	0.54
m2	age * environment + ID (random effect)	~ scale_age * type + (1|ID)	6	1.5	0.25
m4	age + age^2 * environment + ID (random effect)	~ scale_age + I(scaleage^2) * type + (1|ID)	7	2.6	0.15
m3	age + age^2 + environment + ID (random effect)	~ scale_age + I(scaleage^2) + type + (1|ID)	6	4.4	0.06

Supplementary Table 6: Full list of four models run on creatinine-controlled neopterin values (n = 89), models ordered by ΔAICc.

Model	Model description	Model syntax (log creatinine-controlled neopterin ~)	df	ΔAICc	AICc weight
m4	age + age^2 * environment + ID (random effect)	~ scale_age + I(scaleage^2) * type + (1|ID)	7	0	0.95
m2	age * environment + ID (random effect)	~ scale_age * type + (1|ID)	11	6	0.03
m1	age + environment + ID (random effect)	~ scale_age + type + (1|ID)	6	7	0.02
m3	age + age^2 + environment + ID (random effect)	~ scale_age + I(scaleage^2) + type + (1|ID)	5	8.1	< 0.01
